# Commissuroplasty Using Split Dry Lips: A Case Report

**DOI:** 10.7759/cureus.35980

**Published:** 2023-03-10

**Authors:** Takeo Osaki, Saki Kamino, Nobuyuki Murai

**Affiliations:** 1 Plastic Surgery, Hyogo Cancer Center, Akashi, JPN

**Keywords:** dry lip, lip cancer, cross-lip flap, lip reconstruction, commissuroplasty

## Abstract

Commissuroplasty is a procedure that is performed to correct deformities at the corner of the mouth or oral commissure. Herein, we report a case of postoperative microstomia treated with commissuroplasty using split dry lips. In a surgical procedure, the dry lip was divided into orbicularis oris muscle cutaneous flaps and transpositioned into the cleft formed. The deformation of the corners of the mouth improved, and mouth opening improved enough to wear dentures. We believe that this method enables commissuroplasty that combines aesthetics with function.

## Introduction

Commissuroplasty is a procedure used to correct a deformity at the corner of the mouth or the oral commissure. Commissuroplasty may be performed to correct macrostomia resulting from the lateral orofacial cleft. More often, commissuroplasty is performed to repair microstomia as a result of trauma or after reconstruction of lip defects involving the oral commissure [1].

After cross-lip flap surgery such as fan flap, microstomia and deformity at the corner of the mouth may occur, requiring commissuroplasty [2]. However, the lip, especially dry lip, has limited tissues, and it is challenging to perform commissuroplasty with good results in terms of aesthetics and function. Therefore, we present a case of commissuroplasty with good results using split dry lips for microstomia and deformity at the corner of the mouth after upper lip reconstruction.

## Case presentation

**Figure 1:**
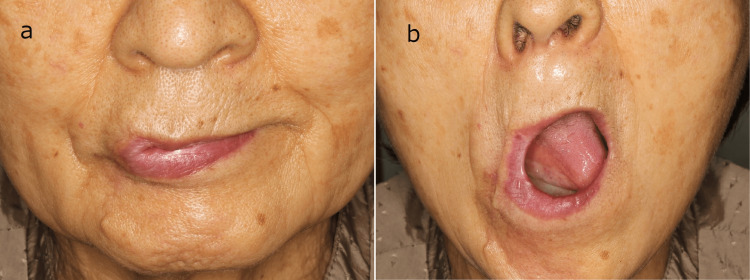
Preoperative photographs of the commissuroplasty procedure (a) Closed-mouth findings: the right corner of the mouth is deformed. (b) Open-mouth findings: the patient cannot wear dentures due to microstomia.

A 76-year-old woman visited our hospital's oral and maxillofacial surgery department with a chief complaint of unhealed stomatitis on the right upper lip. She was diagnosed with squamous cell carcinoma by biopsy and underwent surgery. During the initial operation, the right upper vermilion was resected 3 cm wide from the right corner of the mouth and reconstructed with a local skin flap from the lower right lip. After that, the medial margin was positive, and additional resection and suturing were performed. After the operation, she developed microstomia and deformed mouth corners and was unable to wear dentures (Figure 1).

**Figure 2:**
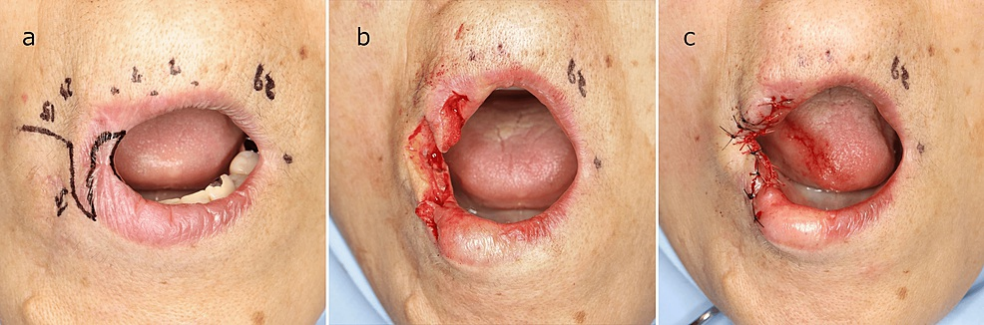
Intraoperative photographs (a) Surgical incision design: the new corner of the mouth was determined by measuring the distance from the cupid's bow on the unaffected side to the corner of the mouth and making a point at the same distance from the cupid's bow on the affected side. (b) Division of the dry lip into orbicularis oris muscle cutaneous flaps. (c) Suturing of each dry lip flap to the mucosa on the oral side.

Six months later, commissuroplasty was performed. The corner of the mouth was incised to a symmetrical position of the corner of the mouth on the unaffected side; this new position of the corner of the mouth was determined by measuring the distance from the cupid's bow on the unaffected side to the corner of the mouth and making a point at the same distance from the cupid's bow on the affected side. The dry lip was divided into orbicularis oris muscle cutaneous flaps and transpositioned into the cleft formed. Each dry lip flap was sutured to the mucosa on the oral side (Figure 2).

**Figure 3:**
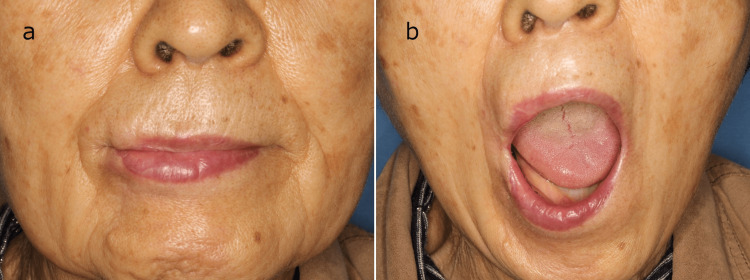
Six months after surgery (a) Closed-mouth findings: improvement in the deformation of the corners of the mouth. (b) Open-mouth findings: improvement in microstomia; the patient is able to wear dentures.

The flap survived, and the wound healed without complications. The operation was successful as the deformation of the corners of the mouth improved, and it became possible to wear dentures. Six months after the operation, no recurrence of the tumor was observed, and no problems were observed in both functional and cosmetic aspects (Figure 3).

## Discussion

Microstomia may occur secondary to lip reconstruction especially with the use of cross-lip flaps that can lead to rounding of the oral commissure [3]. Commissuroplasty techniques for microstomia include tongue flap [4-7], red lip flap [8], and buccal mucosal advancement or myomucosal advancement flap [3,9,10]. Tongue flap is a safe method that provides sufficient volume for large defects. However, this flap often requires a second operation, and the burden on the patient such as restricted eating and pain cannot be neglected. In addition, since the texture of the tongue is different from that of red lip, aesthetic appearance is poor, and dry lips cannot be reconstructed. The vermillion lip advancement flap is a method that can reconstruct a dry lip, but it is difficult when the width of the defect is 1.5 cm or more [8]. The buccal mucosal advancement flap is often used when the defect is large, but this method also cannot reconstruct the dry lip.

Dry lips are essential for natural and functional lips. Several reports show that a dry lip defect was reconstructed with the remaining dry lip. Kawamoto described corrections to major lower red lip defects by using some areas of the upper dry lip [11]. Haramoto et al. reconstructed the lower red lip defect using the residual dry lip as a local flap [12]. Suda et al. reported a method of reconstructing a half red lip defect using the remaining red lip [13]. The common ground is that as much as possible, dry lips should be reconstructed using dry lips. So, in this case, we attempted to reconstruct both the upper and lower dry lips using a split dry lip in commissuroplasty. In addition to the advantage of dry lip reconstruction on the upper and lower lips, this method provides good contours. The large Z plasty flap makes the commissure red lip thin and resembles the form of a normal commissure.

This method, however, has a limitation. The dry lip inevitably has a narrow width, and the skin flap becomes thin when stretched. It was thought that there would be concerns about blood circulation. In this case, the dry lip did not reach the corners of the lower lip because the flap, if stretched too thin, would undergo necrosis. However, there were no issues with aesthetics and function.

## Conclusions

We reported a method of commissuroplasty using split dry lips. Although there are limitations on the length and width of the flap, we believe that this method enables commissuroplasty that combines aesthetics and function.
